# Dual-axis control of magnetic anisotropy in a single crystal Co_2_MnSi thin film through piezo-voltage-induced strain

**DOI:** 10.1039/d1na00864a

**Published:** 2022-05-26

**Authors:** Bao Zhang, Siwei Mao, Chunlong Li, Peizhen Hong, Jingwen Hou, Jianhua Zhao, Zongliang Huo

**Affiliations:** Institute of Microelectronics, Chinese Academy of Sciences 100029 Beijing China huozongliang@ime.ac.cn; State Key Laboratory of Superlattices and Microstructures, Institute of Semiconductors, Chinese Academy of Sciences Beijing 100083 China; Center of Materials Science and Optoelectronics Engineering, University of Chinese Academy of Sciences Beijing 100190 China; College of Microelectronics, University of Chinese Academy of Sciences 100049 Beijing China lichunlong@ime.ac.cn; Yangtze Memory Technologies Co., Ltd (YMTC) 430205 Wuhan China

## Abstract

Voltage controlled magnetic anisotropy (VCMA) has been considered as an effective method in traditional magnetic devices with lower power consumption. In this article, we have investigated the dual-axis control of magnetic anisotropy in Co_2_MnSi/GaAs/PZT hybrid heterostructures through piezo-voltage-induced strain using longitudinal magneto-optical Kerr effect (LMOKE) microscopy. The major modification of in-plane magnetic anisotropy of the Co_2_MnSi thin film is controlled obviously by the piezo-voltages of the lead zirconate titanate (PZT) piezotransducer, accompanied by the coercivity field and magnetocrystalline anisotropy significantly manipulated. Because in-plane cubic magnetic anisotropy and uniaxial magnetic anisotropy coexist in the Co_2_MnSi thin film, the initial double easy axes of cubic split to an easiest axis (square loop) and an easier axis (two-step loop). While the stress direction is parallel to the [1−10] easiest axis (sample I), the square loop of the [1−10] direction could transform to a two-step loop under the negative piezo-voltages (compressed state). At the same time, the initial two-step loop of the [110] axis simultaneously changes to a square loop (the easiest axis). Otherwise, we designed and fabricated the sample II in which the PZT stress is parallel to the [110] two-step axis. The phenomenon of VCMA was also obtained along the [110] and [1−10] directions. However, the manipulated results of sample II were in contrast to those of the sample I under the piezo-voltages. Thus, an effective dual-axis regulation of the in-plane magnetization rotation was demonstrated in this work. Such a finding proposes a more optimized method for the magnetic logic gates and memories based on voltage-controlled magnetic anisotropy in the future.

## Abstract 2

Dual-axis controlled magnetic anisotropy and magnetization 90° rotation are demonstrated in Co_2_MnSi/GaAs/PZT heterostructures under piezostrain. The periodic response of magnetization is achieved under the pulsed piezo-voltages of ±40 V.

## Graphical abstract

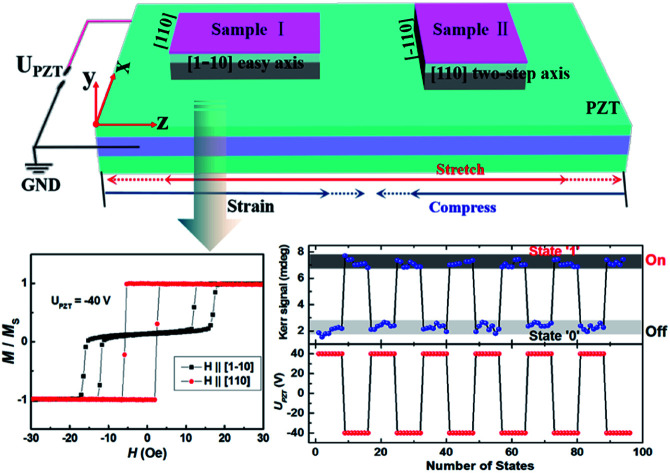

## Introduction

Pure electrical manipulation of magnetization rotation in magnetic devices is a desirable way for spintronic applications, which is suitable for the scaling of devices in the integrated circuit. To date, there have been multiple types of electrical manipulation ways of the magnetization rotation or magnetic anisotropy variation, such as spin–orbit torque (SOT),^1–7^ spin transfer torque (STT),^8–11^ magneto-electrical coupling (MEC) effect,^12–17^ and strain.^18–25^ The piezo-voltage-induced strain, as a way of voltage-controlled magnetic anisotropy (VCMA), has attracted the attention of many researchers due to the performance of low-power consumption.^22,25^ The main studies of strain controlled magnetization are related to the inverse piezoelectric effect of piezoelectric materials in the ferromagnetic/piezoelectric heterostructure. The magneto-crystalline anisotropy is controlled by voltage through the piezo-voltage-induced strain transformed to the magnetic thin film. Usually, the change of magnetocrystalline anisotropies is related to the strain-manipulated variations of the lattice constant.^23^ However, the previous relative studies of strain-controlled magnetization rotation were mainly demonstrated in a uniaxial regulation manner.^22–24,26^ The stress regulation characteristic disappears or weakens when the specific crystal orientation of magnetic thin films rotates relative to the stress axis. This problem would be effectively avoided by realizing dual-axis or multi-axis stress-regulated magnetization rotation. However, the related research is still relatively lacking.

In recent years, the Co-based full-Heusler alloys have attracted considerable interest due to the high spin polarization and Curie temperature,^27^ which are promising candidates for the next generation information processing and storage in spintronic devices. The coexistence of the in-plane cubic and uniaxial magnetic anisotropies was observed when Heusler alloys are deposited on the GaAs (001) substrate.^28–30^ The initial in-plane easiest axis (square loop) and easier axis (two-step loop) have been measured along two axes of minimum value of the cubic anisotropy, which is caused by the superposition of the uniaxial anisotropy. It is well known that a uniaxial stress could induce an extra uniaxial anisotropy in magnetic films. Thus, using the competition of uniaxial anisotropy induced by interface and stress in magnetic films can realize the regulation of magnetic anisotropy energy and magnetization rotation.

In this work, we have studied the dual-axis control of magnetic anisotropy in the Co_2_MnSi/GaAs/PZT heterostructures through piezo-voltage-induced strain. By studying the variation of the magnetic coercivity (*H*_c_) and remnant magnetization (*M*_r_) in Co_2_MnSi magnetic thin film, the strong voltage-controlled magnetic anisotropy was verified. Furthermore, we measured the periodic-strain controlled magnetization by applying pulsed piezo-voltages. Two stable states have been achieved with the periodic measurement in two samples. The dual-axis control of the magnetic anisotropy in this work proposes a method of voltage-controlled magnetic logic devices, which will simplify the growth process of magnetic materials and reduce energy consumption.

## Methods

**Fig. 1:**
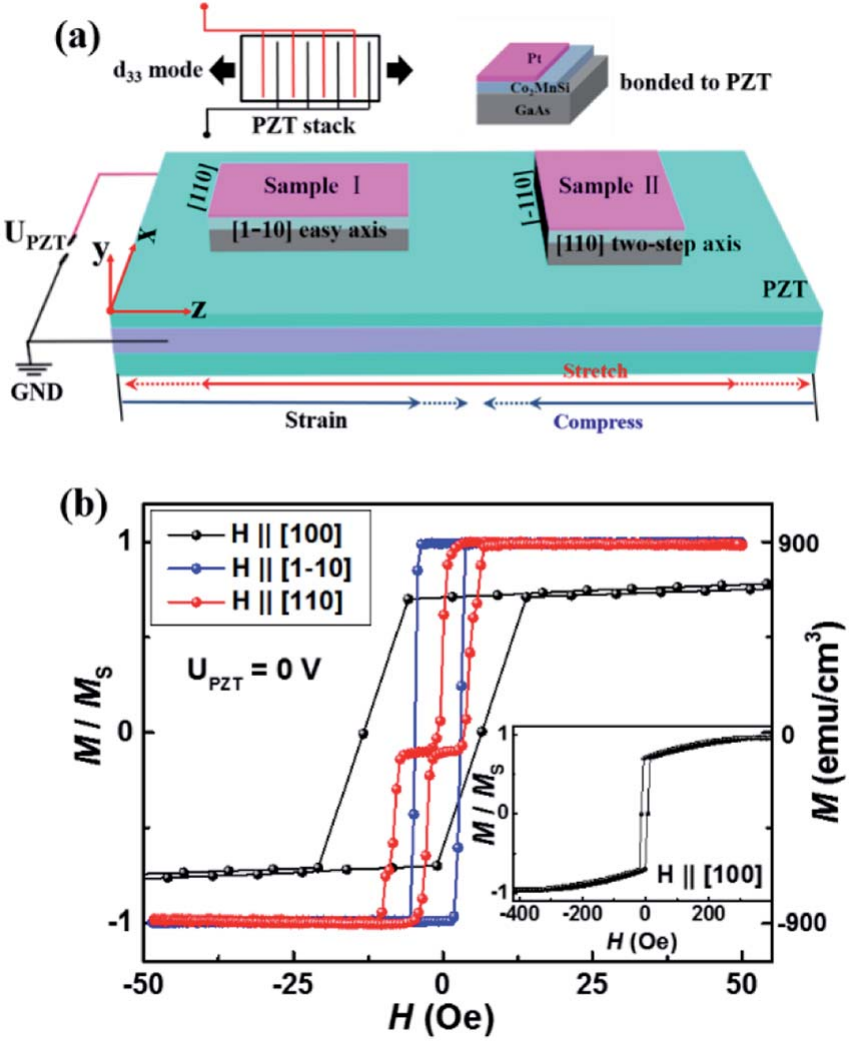
(a) The schematic diagram of Co_2_MnSi/GaAs/PZT heterostructures controlled by piezo-voltages. When the piezo-voltages were applied, the direction of the strain is parallel to the *z*-axis. The [1−10] and [110] directions are parallel to the *z*-axis in samples I and II, respectively. The inset figures are the schematic of an axially acting multilayer piezo-stack and the structure of the magnetic film. (b) The magnetic hysteresis loops of the Co_2_MnSi thin film in the initial state along the in-plane [110], [1−10], and [100] directions. The inset shows the saturated magnetization in the [100] direction with 420 Oe magnetic field.

The 10 nm Co_2_MnSi thin film was grown at 250 °C on a GaAs (001) substrate using molecular-beam epitaxy (MBE).^28,29^ Its Curie temperature is 985 K.^27^ Following the growth of 10 nm-thick single crystal Co_2_MnSi, a 3 nm thick platinum (Pt) layer was deposited to avoid the oxidation (as shown in Fig. 1a). In order to guarantee that the strain induced by the lead zirconate titanate (PZT) piezotransducer can be effectively transferred to the Co_2_MnSi film, we thinned the GaAs substrate of the sample to 100 μm before it was bonded to PZT by two-component epoxy. To study the dual-axis control of magnetic anisotropy, the [1−10] and [110] directions of Co_2_MnSi samples are parallel to the *z*-axis of PZT, respectively. The Co_2_MnSi/GaAs/PZT heterostructure was in a compressed state when the piezo-voltage was negative, and in a stretched state when the piezo-voltage was positive (as shown in Fig. 1a). The magnitude of the additional uniaxial strain for a piezo-voltage of 80 V is approximately 5.2 × 10^−4^.^30^ The magnetization vectors of the Co_2_MnSi samples (*S* = 3 × 4 mm^2^) were measured by longitudinal magneto-optical Kerr microscopy (Nano MOKE3) and a superconducting quantum interference device (SQUID) magnetometer. The piezo-voltages were applied with an Agilent B1500A with the leading and trailing time being both 100 ns. All the measurements were carried out at room temperature.

## Results and discussion

Fig. 1a shows the schematic diagram of Co_2_MnSi/GaAs/PZT heterostructures controlled by piezo-voltage-induced strain. Two samples of Co_2_MnSi/GaAs are bonded to the PZT, where the [1−10] and [110] crystal orientations are parallel to the *z*-axis in samples I and II, respectively. The piezoelectric ceramic blocks are stacked along the *z* direction, and the reverse piezoelectric effect appears in the d_33_ mode (the inset figure shows the schematic of an axially acting multilayer piezo-stack). When the positive and negative piezo-voltages (*U*_PZT_) are applied, the PZT would exhibit a tensile and compressive strain, respectively.^23^ Under the stress regulation, the samples I and II will undergo different regulation effects. Fig. 1b shows the initial magnetic hysteresis loops of the Co_2_MnSi thin film along the [110], [1−10] and [100] directions. The saturation magnetization *M*_s_ is about 884 emu cm^−3^. The [1−10] orientation is an easy axis with a square loop and the [110] orientation is a two-step loop with *M*_r_ = 0 when the magnetic field is zero. The [100] orientation is a hard axis with a 400 Oe saturation field (the saturated magnetized state in the [100] direction is shown in the inset of Fig. 1b).

**Fig. 2:**
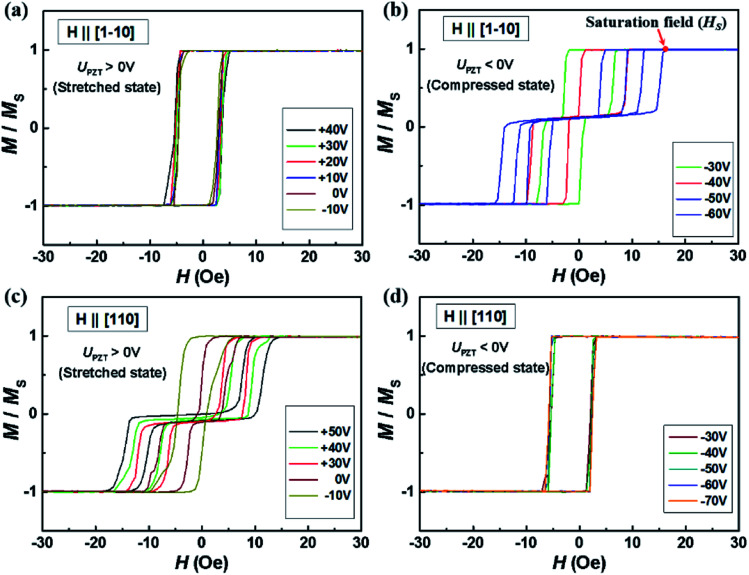
The magnetic hysteresis loops under different piezo-voltages (from −60 V to 50 V; step is 10 V) along the in-plane [1−10] and [110] directions. (a) The magnetic hysteresis loops in the [1−10] direction are square curves with *U*_PZT_ > −10 V. (b) Contrary to (a), the square curve changed to two-step curves with *U*_PZT_ < −30 V and the saturated field increased with the increase of *U*_PZT_ absolute value. (c) The magnetic hysteresis loops in the [110] direction are changed to two-step curves from square curves with *U*_PZT_ > 0 V. (d) The magnetic hysteresis loops keep the square curve with *U*_PZT_ < −30 V (compressed states).

**Fig. 3:**
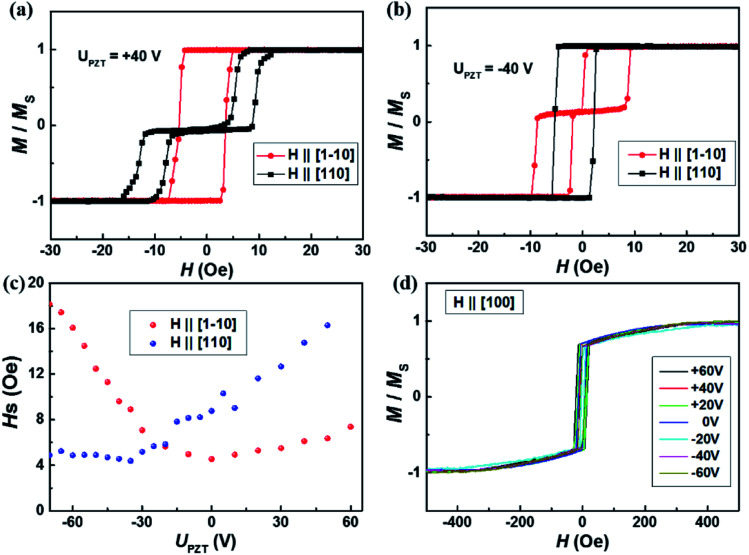
The magnetic properties of the Co_2_MnSi film under different piezo-voltages. (a) Compared with the Co_2_MnSi film under different piezo-voltages, the magnetic hysteresis loops of [1−10] and [110] directions keep square and two-step curves with *U*_PZT_ = 40 V (stretched states), respectively. (b) The two-step axis and the square axis are exchanged with *U*_PZT_ = −40 V (compressed states). (c) The piezo-voltage dependence of the saturation field in the [1−10] and [110] directions; the voltage step is 5 V. (d) The magnetic hysteresis loops maintain the hard axis in the [100] direction under different piezo voltages.

To further study the dual-axis control of magnetic anisotropy in Co_2_MnSi/GaAs/PZT heterostructures, we measured the magnetic hysteresis loops of two samples along the [1−10], [110] and [100] directions under positive and negative piezo-voltages. Fig. 2 shows the piezo-voltage controlled magnetic hysteresis loops in sample I. With the piezo-voltages increasing from −10 to 40 V, the loops of the [1−10] direction kept stabilized (as shown in Fig. 2a). At the same time, we also measured the loops of the [110] direction and the loops changed to a two-step loop, accompanied by increasing the saturation field from 6.1 to 13.1 Oe (as shown in Fig. 2c). From there, the stretched strain could effectively manipulate the in-plane magnetocrystalline anisotropy. In contrast, we also measured the loops compression state under negative piezo-voltages. The magnetic hysteresis loops of the [1−10] easy axis showed obvious regulatory phenomena (as shown in Fig. 2b). With the piezo-voltages changing from −30 to −60 V, the saturation field of the two-step loop increased from 7.2 to 16.5 Oe. However, the loop of the [110] axis changed to a square curve and kept stabilized (as shown in Fig. 2d). Through the regulation of piezo-voltages, we can obtain two completely opposite and stable phenomena under ±40 V (as shown in Fig. 3a and b), which would be able to meet the needs of two states and facilitate the design of magnetic storage and logic devices. In order to clarify the discipline of VCMA in sample I, the dependence of the saturation field with piezo-voltages along the [110] and [1−10] directions is summarized in Fig. 3c. Obviously, the saturation field changes gradually with the piezo-voltage and the variation trend of the saturation field is exactly opposite in the [110] and [1−10] directions. It indicates that the in-plane magnetocrystalline anisotropy of the Co_2_MnSi film is obviously controlled under the regulation of piezo-voltages. In addition, we also measured the magnetic hysteresis loops along the [100] crystal direction (as shown in Fig. 3d). The loops of the [100] direction keep a hard axis loop with a slight change of coercive field under positive or negative piezo-voltages. Through the study of piezo-voltage controlled in-plane crystalline anisotropy in sample I, the VCMA is well demonstrated in the Co_2_MnSi/GaAs/PZT heterostructures.

**Fig. 4:**
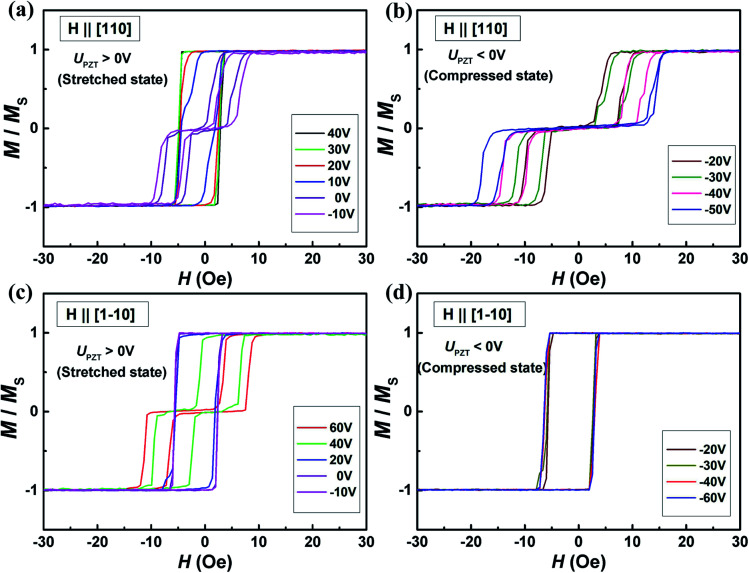
The magnetic hysteresis loops under different piezo-voltages (from −40 V to 90 V, step is 10 V) along the in-plane [110] and [1−10] directions of the sample II. (a) The magnetic hysteresis loops in the [110] direction change to square curves from two-step curves with *U*_PZT_ > 20 V (stretched states). (b) The magnetic hysteresis loops in the [110] direction keep the two-step curves with *U*_PZT_ < −20 V and the saturation field increase with the negative piezo-voltage increase. (c) The magnetic hysteresis loops in the [1−10] direction are changed to two-step curves from square curves with *U*_PZT_ > 40 V. (d) The magnetic hysteresis loops keep the square curve with *U*_PZT_ < −20 V in the [1−10] direction.

**Fig. 5:**
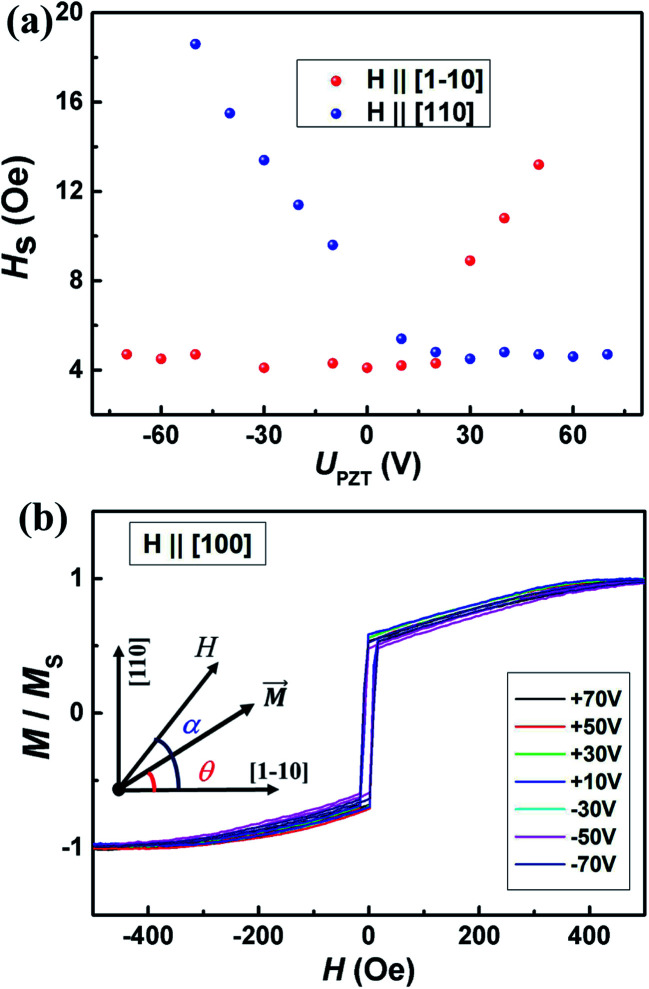
The magnetic properties of the Co_2_MnSi film under different piezo-voltages. (a) The piezo-voltage dependence of the saturation field in the [1−10] and [110] directions; the voltage step is 10 V. (b) The magnetic hysteresis loops maintain the hard axis in the [100] direction under different piezo-voltages. The inset is the definition of the *θ* and *α*. *θ*(*α*) is the angle between the magnetization (magnetic field) and [1−10] direction.

In order to verify the dual-axis control of magnetic anisotropy in the Co_2_MnSi thin film, we also studied the VCMA in sample II. Similar to the measurement of sample I, we have detected the variations of the magnetic hysteresis loops along the [110], [1−10] and [100] directions under positive and negative piezo-voltages. Fig. 4a and b show the loops of the [110] crystal direction with the piezo-voltages. With the stress parallel to the [110] direction, the manipulation phenomenon of VCMA is obvious. When we applied a positive piezo-voltage to the PZT, the loop of the [110] direction changes to a square curve from a two-step loop, which indicates that [110] has been converted to an easy magnetized axis. However, the [110] direction keeps a two-step loop under the negative piezo-voltages (compressed state) (as shown in Fig. 4b). The saturation field increased with the piezo-voltages changing from −20 to −50 V. In order to analyze the variation of magnetocrystalline anisotropy, we measured the magnetic hysteresis loop of the [1−10] direction under positive and negative piezo-voltages. In contrast to sample I, the magnetic hysteresis loops of the [1−10] varied from a square curve to a two-step loop with the piezo-voltage increasing from −10 to 60 V (as shown in Fig. 4c). Under the negative piezo-voltages, the loops of the [1−10] direction keep the square curve stabilized (as shown in Fig. 4d). We also measured the magnetic hysteresis loops of the [100] hard magnetic axis and the loops maintained the hard magnetic properties with the positive or negative piezo-voltages (only a part of the loops shown in Fig. 5b). Through the magnetic measurement along different directions, we summarized the variation of the saturated field with the piezo-voltage in Fig. 5a. The saturated fields of [110] and [1−10] directions show opposite trends with the piezo-voltages. When the piezo-voltage increases to 40 V, the [1−10] direction has a large saturated field and shows a two-step loop. When the piezo-voltages changed from −10 to +20 V, the [110] direction varied to a two-step loop and the [1−10] direction varied to a square loop (easiest magnetization axis). From the measurement of sample II, we once again demonstrated the piezo-voltage control of magnetocrystalline anisotropy in the Co_2_MnSi/GaAs/PZT heterostructures.

Through the demonstration of dual-axis control of magnetic anisotropy in epitaxial Co_2_MnSi thin films through piezo-voltage-induced strain, we could achieve a purely electrical controlled magnetization rotation. The magnetization rotation could attribute to an extra in-plane uniaxial anisotropy induced by the piezo-voltage in the Co_2_MnSi thin films. In order to quantify the magnetocrystalline anisotropy of Co_2_MnSi thin films with the piezo-voltages, the magnetic anisotropy energy *E* can be described as^31^1
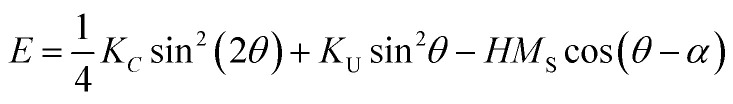
where *K*_U_ and *K*_C_ are the effective uniaxial and cubic anisotropy constants respectively, *H* is the applied external magnetic field, *M*_S_ is the saturation magnetization, *θ* is the angle between the magnetization and the easiest axis [1−10], and *α* is the angle between the external magnetic field and the easiest axis [1−10] (see the inset of Fig. 5b). With the saturation field and slope of the two-step loops, the *K*_U_ and *K*_C_ can be calculated as2
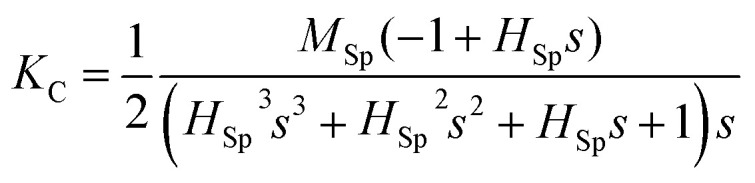
3
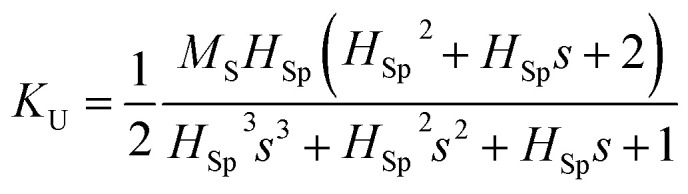
where *H*_Sp_ is the so-called split field and *s* is the constant slope between *H*_Sp_ and −*H*_Sp_.^31^ In this work, we calculated the values of *K*_U_ and *K*_C_ under ±40 V piezo-voltages in sample I. The *K*_U_ and *K*_C_ are obtained to be 1.28 ± 0.06 kJ m^−3^ and 17.69 ± 0.88 kJ m^−3^ with a piezo-voltage of 40 V, respectively. When the piezo-voltage is −40 V, the *K*_U_ and *K*_C_ transform to −0.83 ± 0.04 kJ m^−3^ and 10.64 ± 0.53 kJ m^−3^, respectively. Obviously, the easiest magnetization axis is changed from [1−10] (*U*_PZT_ = 40 V) to [110] (*U*_PZT_ = −40 V) direction. For sample II, the values of *K*_U_ and *K*_C_ are also obtained to be −0.91 ± 0.05 kJ m^−3^ (0.77 ± 0.04 kJ m^−3^) and 12.42 ± 0.62 kJ m^−3^ (18.62 ± 0.93 kJ m^−3^) under a piezo-voltage of 40 V (−40 V), which also induced the transformation of the easiest magnetization axis and magnetization 90° rotation.

**Fig. 6:**
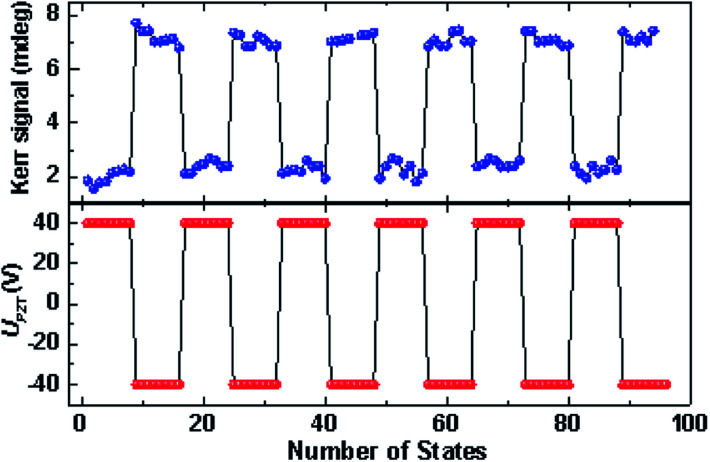
The periodic changes of the magnetization (shown as Kerr signal) in sample I with the periodic change of the piezo-voltage between −40 V and 40 V without the external magnetic field.

Based on the demonstration of dual-axis control of magnetization rotation in Co_2_MnSi/GaAs/PZT heterostructures, it will be promising to be applied in the design of magnetic functional devices, such as magnetic tunneling junction (MTJ) and planar Hall devices. To further study the response performance of the Co_2_MnSi/GaAs/PZT device, we measured the magnetization during the periodic change of piezo-voltage between −40 and 40 V in sample I without the external magnetic field. The magnetization periodically switched from 7.5 to 2.3 mdeg, which correspond to the ‘1’ and ‘0’ states of the logic device (as shown in Fig. 6). The time response of the piezo-voltage controlled device has been investigated, where the rising and falling time are 361.7 μs and 376.2 μs, respectively. Therefore, we could utilize voltage control of magnetization rotation to design and fabricate the magnetic logical arrays to realize the information processing in Heusler alloys. Our study identified that the dual-axis control of magnetization rotation through strain engineering could have a great prospect for spintronic applications.

## Conclusions

In summary, we have demonstrated the dual-axis (the [1−10] and [110] directions) control of magnetic anisotropy in epitaxial Co_2_MnSi thin films through piezo-voltage induced strain. Furthermore, we demonstrated the periodically voltage-controlled magnetization rotation in the Co_2_MnSi/GaAs/PZT heterostructure. Under applied piezo-voltages, the in-plane magnetization rotation could be implemented without extra magnetic field. The piezo-voltage-induced strain is the primary mechanism in the Co_2_MnSi/GaAs/PZT heterostructure, which induces an extra uniaxial anisotropy along the in-plane crystalline orientation of the Co_2_MnSi film and manipulates the direction of the minimal anisotropy energy. Compared with the uniaxial control effect of many magnetic materials, the dual-axis control of Co_2_MnSi could be manipulated effectively through the strain and is more suitable for the magnetic logic devices. This result will pave the way to the design and fabrication of dual-axis control of spintronic devices based on the voltage-controlled magnetic anisotropy.

## Author contributions

B. Z. and C. L. conceived the work. S. M. performed the sample growth. B. Z. fabricated the devices and performed the experiments. B. Z., C. L., P. H. and Z. H. analyzed the data. B. Z., C. L. J. Z. and Z. H. wrote the manuscript. All authors discussed the results and commented on the manuscript.

## Conflicts of interest

There are no conflicts to declare.

## Supplementary Material
